# Rationale and design of a pragmatic clinical trial to assess the impact of self-monitoring blood pressure at home and self-titration of antihypertensive medication in poorly controlled hypertension: the ADAMPA study protocol

**DOI:** 10.1186/s12875-018-0846-y

**Published:** 2018-09-24

**Authors:** José Sanfélix-Genovés, Clara L. Rodríguez-Bernal, Irene Marco-Moreno, Patricia Martinez-Ibañez, Lucía Martinez-Ibañez, María Bóveda-García, Ignacio Barreira-Franch, Mercedes Calleja-Del Ser, Greta Borrás-Moreno, Eugenia Avelino-Hidalgo, Marina Escrig-Veses, Margherita Lauriano, Margarita Giménez-Loreiro, Laura Bellot-Pujalte, Aníbal García-Sempere, Salvador Peiró, Gabriel Sanfélix-Gimeno, ADAMPA group, Joaquín Abad-Carrasco, Maria V. Agudo-Escagüés, Marta Alvarez-Martinez, Rosa M. Bartual-Penella, Rosa Carrión-Villanueva, Encarnación Checa-Sanz, Ana Costa-Alcaraz, Isabel Cristófol-López, Aurelio Duque-Valencia, Rosario González-Candelas, Ricardo González-Espadas, Luis González-Luján, Victoria Gosalbes, Enrique Guinot-Martínez, Emilio L. López-Torres, Silvia Molla-LLosa, Víctor Moreno-Comins, Miriam Moreno-Prat, María J. Puchades-Company, Ángela Ramos-García, Paloma Ramos-Ruiz, Ester Robles-Pastor, Pilar Roca-Navarro, Rosa Saiz-Rodriguez, Julia L. Salanova-Chilet, Ana Tchang-Sanchez, Francisca Torres, Ruth Uribes-Fillol, Cristina Valle-García, Macarena Villar-Ruiz, Cristina Vivas-Miquel

**Affiliations:** 1 Centro de Salud de Nazaret, Departamento de Salud de Valencia Clínic-La Malvarrosa, Valencia, Spain; 2 Health Services Research Unit, FISABIO, Valencia, Spain; 3 Spanish Network of Chronic Care and Health Services Research (REDISSEC), Valencia, Spain; 4 Health Research Institute (INCLIVA), Valencia, Spain

**Keywords:** Self-monitoring, Blood pressure, Hypertension, Self-titration, Primary care, Pragmatic clinical trial

## Abstract

**Background:**

Lack of control of hypertension is one of the most prevalent problems encountered by general practitioners (GPs). Self-measured blood pressure monitoring at home (SMBP) and self-titration of medication could be a good strategy to improve hypertension management, however, evidence is limited and not conclusive. We aimed to assess the effectiveness, in the primary care setting, of an intervention that includes educational components, SMBP and self-titration of antihypertensive medication to decrease systolic blood pressure compared to usual care, in a population with poorly controlled hypertension, during a 12-month period.

**Methods:**

Pragmatic, controlled, randomized, unblinded clinical trial with two parallel groups assigned in a ratio of 1:1 to self-management (which includes educational components, SBMP and self-titration of antihypertensive medication based on a patient’s GP’s pre-established adjustment plan) or to usual care (with educational components too).

**Discussion:**

If the data from this trial show positive results, the study may contribute to a change of strategy in the treatment of hypertension, focusing on the patient as the main actor to achieve blood pressure control. Furthermore, this approach might contribute to the financial sustainability of the National Health Service.

**Trial registration:**

This trial has been registered in the database with reference number EudraCT: 2016-003986-25. Registered 05 May 2017, https://www.clinicaltrialsregister.eu/ctr-search/search?query=2016-003986-25

## Background

The presence of hypertension is one of the most important issues in the global burden of disease [1]. In developed countries, the degree of control of hypertension has increased progressively over the last 15 years and has contributed to a decline in cardiovascular morbidity and mortality [2–8]. However, a recent study carried out in 12 European countries showed that more than 50% of patients treated for hypertension continued to have uncontrolled blood pressure (BP) [9] and that results are far from ideal. As a large part of hypertension management is carried out in primary care (PC) and it is one of the most prevalent problems encountered by General Practitioners (GP), interventions aimed at improving its management should preferably be made in this setting. Recent hypertension clinical guidelines put emphasis on self-measured blood pressure monitoring (SMBP) by patients and on team-based systems to manage the condition [10].

Self-measured blood pressure monitoring at home (SMBP) is practiced extensively nowadays. In the United Kingdom and Canada it is highly recommended by GPs and used by more than 30% of patients [11, 12]. Systematic reviews have shown disparate information regarding the effectiveness of SMBP alone in reducing blood pressure (BP). On the other hand, self-monitoring in conjunction with co-interventions (including systematic medication titration by doctors, pharmacists, or patients; education; or lifestyle counseling) has been shown to lead to clinically significant BP reduction, which persists for at least 12 months. Nevertheless, the effectiveness of SMBP requires additional evaluation given that its definition in those studies is highly heterogeneous (different clinical protocols, different strategies for additional support and management) and given the fact that most studies have short follow-ups (1 year or less) [13, 14].

Regarding home titration of antihypertensive medication, evidence is more limited and shows mixed results. Two clinical essays, the TASMINH2 [15] and the TASMINH-SR [16], both in the United Kingdom and developed in the primary care setting by the same research team, are some of the most recent and interesting clinical trials carried out in this field. In these studies, SMBP together with self-titrate medications (according to a previously agreed plan), combined with telemedicine components, was compared with usual care. In both studies systolic blood pressure (SBP) decreased from baseline to 12 months, with significant differences between the intervention and control group (5.4 and 9.2 mmHg, respectively). Frequency of side effects was similar in both groups [15, 16]. The TASMINH-SR study is of special relevance because it was carried out with high risk patients (with a personal history of stroke, ischemic cardiopathy, diabetes or kidney failure), a population of special interest to achieve BP targets [16]. On the other hand, a clinical trial carried out in the US in a low-income, predominantly minority population, aimed to determine whether health coaching, SMBP and home titration of antihypertensive medications could improve BP control compared with SMBP and health coaching alone. The results showed that both the home-titration arm and the no–home-titration arm had a reduction in SBP, with no significant differences between them from baseline to 6 months [17].

Finally, when interpreting hypertension studies over time, it is important to procede with caution, as the definition of the condition changes almost with every update of guidance. For instance, earlier versions of guidelines such as those of the the Joint National Committee (JNC) and of the European Society of Hypertension (ESH)/European Society of Cardiology (ESC), suggested more restrictive BP control objectives than recent versions (especially in patients over 60 years old, diabetics and patients with renal failure) [18–20]. These objectives may be modified again in the light of the results of recent studies [21–23].

### Study aim

The primary aim of the ADAMPA TRIAL is to assess the effectiveness, in the primary care setting, of an intervention that includes educational components, SMBP and self-titration of antihypertensive medication to decrease SBP compared to usual care, in a population with poorly controlled hypertension, during a 12-month period. In addition, an extension with passive follow-up is planned for 24 months, collecting a reduced set of outcome variables as secondary variables.

### Main research questions

1. Does a self-management intervention based on SMBP and self-titration medication allow for better control of blood pressure?
2. What is the impact of this intervention on adherence to treatments, lifestyle changes, quality of life, clinical outcomes and use of health services?
3. Is this intervention cost-effective?
4. What are patients’, caregivers’ and health professionals’ views and experiences of SMBP plus self-titration in poorly controlled hypertension?

## Methods

### Study design and setting

The ADAMPA study is a pragmatic, controlled, randomized, unblinded clinical trial with two parallel groups assigned in a ratio of 1:1 to self-management (which includes educational components, SBMP and self-titration of antihypertensive medication based on a patient’s GP’s pre-established adjustment plan) or to usual care (with educational components too).

All participants belong to a Health Area in the Valencia Region (Spain), with a population coverage of 345,500 inhabitants and a geographical area covering the north-east of the city of Valencia and some surrounding towns that are served by sixteen Primary Care Centers (PCC), two Hospitals and a Medical Specialty Centre.

This trial has been registered in the https://eudract.ema.europa.eu/ database with reference number EudraCT: 2016–003986-25.

### Study participants

#### Identification and recruitment

Potential patients eligible to participate in the study will be selected by their General Practitioners (GP) among all patients attending their general practice (case-finding). In their general practice at the PCC, the GPs will inform patients of the study objective and in the case that they meet the inclusion criteria and none of exclusion criteria, GPs will give them the information sheet and informed consent form, responding to all queries and concerns.

#### Eligibility criteria

Eligibility criteria will aim to recruit patients with treated hypertension managed in primary care, who are aged 40 years or older, have a diagnosis of hypertension in their electronic history (coded), have a mean BP reading on the reference arm of SBP > 145 or diastolic blood pressure (DBP) > 90 mmHg on the baseline examination, who voluntarily accept participation in the study and sign the corresponding informed consent. The mean BP will be obtained as follows: In the first visit, BP should be measured on both arms. If there are differences, the reference arm should be that with the highest value of BP. Subsequently, the average BP of at least two measurements, in the sitting position, spaced 1–2 min apart should be calculated. If the first two readings are very different, an additional measurement should be done and the mean BP will be the average of the two readings considered valid [8].

#### Exclusion criteria

Exclusion criteria will include inability to self-manage their BP, including dementia or significant cognitive impairment (at the discretion of the researcher performing the recruitment), a history of orthostatic hypotension (fall> 20 mmHg from SBP after taking the orthostatic position), SBP > 200 or DBP > 100 mmHg in the baseline examination, being prescribed more than 4 antihypertensive drugs, inclusion in another hypertension study or clinical trial, presence of tremor or neurological disease that makes it difficult to perform SMBP, presence of arrhythmia, presence of terminal illness, chronic incapacitation to leave home, an acute cardiovascular event in the last 3 months, hypertension managed directly by other specialist doctors outside the primary care environment, pregnant women or those actively seeking to become pregnant, having a household member already enrolled in the study and non- or temporary residents.

### Randomization

Patients with uncontrolled hypertension will be randomized in a 1:1 ratio to receive either usual care or self-management using a computer randomization system via internet. Minimization will be used [24], taking into account age, gender, SBP > 160 mm HG, diabetes, cardiovascular disease (ischemic heart disease, heart failure, cardiomyopathy and peripheral arterial disease), stroke (chronic stroke) and chronic kidney disease status. Similar approaches have been used in previous clinical trials of self-monitoring in the control of hypertension [15, 16].

### Participant flow through the study

**Fig. 1:**
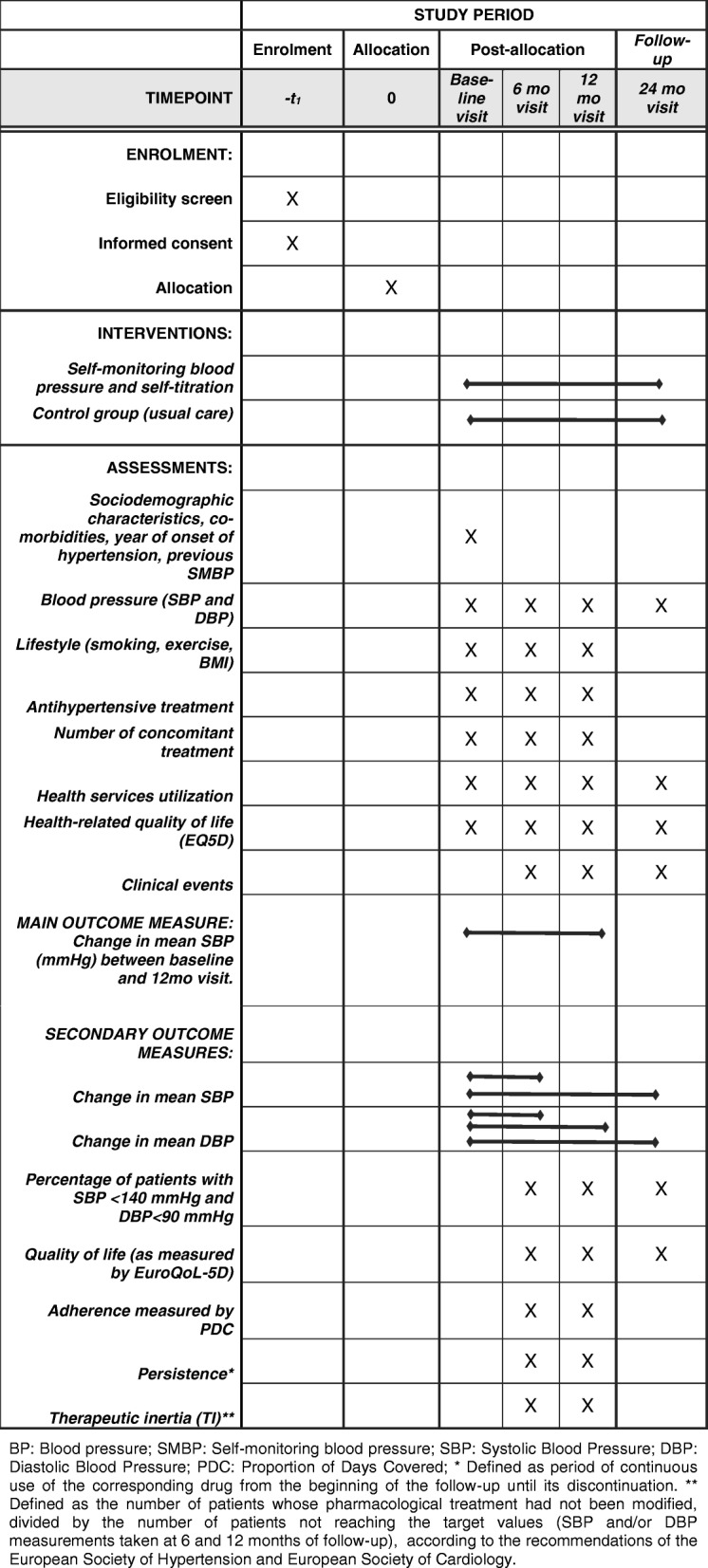
SPIRIT flow diagram: Schedule of enrolment, interventions, and assessments in the ADAMPA study. *BP* Blood pressure, *SMBP* Self-monitoring blood pressure, *SBP* Systolic Blood Pressure, *DBP* Diastolic Blood Pressure, *PDC* Proportion of Days Covered. * Defined as period of continuous use of the corresponding drug from the beginning of the follow-up until its discontinuation. ** Defined as the number of patients whose pharmacological treatment had not been modified, divided by the number of patients not reaching the target values (SBP and/or DBP measurements taken at 6 and 12 months of follow-up), according to the recommendations of the European Society of Hypertension and European Society of Cardiology

An overview of the schedule of enrolment, interventions, and assessments in the ADAMPA study, according to the SPIRIT guidelines is shown in Fig. 1**.** Each aspect will be described in more detail throughout the present protocol.

Recruited hypertensive patients who meet the inclusion criteria and none of the exclusion criteria, who have been duly informed (by their GP) of the characteristics of the study, have signed the informed consent and been randomly assigned to the intervention or control group, will proceed as follows:

#### Intervention group

**Fig. 2:**
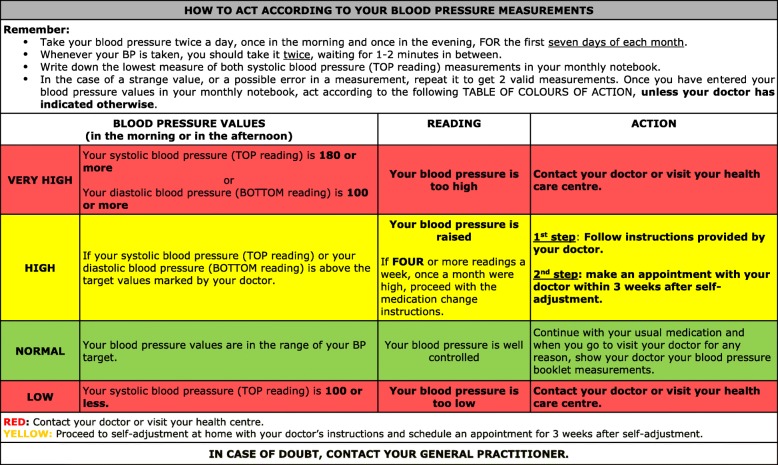
Instructions to patients: “HOW TO ACT ACCORDING TO YOUR BLOOD PRESSURE MEASUREMENTS” in the ADAMPA study. Adapted and modified from: The Colour Coding Chart. Supplementary webappendix in: McManus RJ, Mant J, Bray EP, et al. Telemonitoring and self-management in the control of hypertension (TASMINH2): a randomised controlledtrial. Lancet 2010; published online July 8. DOI:10.1016/S0140-6736(10)60964-6

At their practice, the GPs will have established, with each patient in the intervention group, the BP target according to the European Guide for the management of Hypertension 2013 [19] and how to act according to their BP measurements (Fig. 2), including instructions for medication self-adjustment (if necessary). At the same time, the GP will inform them that they will be recalled to make an appointment with a member of the research team, who will provide them with additional information about their self-management of BP and for completing data corresponding to the baseline visit.

Subsequently, patients will be given -and trained in the use of- a validated home blood pressure monitor (Omron M3 model HEM-7131-E), as well as the “Intervention group booklet” containing: the patient’s code, the reference arm on which BP is measured, the BP target, general information and basic recommendations for improving BP control, instructions to manage the home blood pressure monitor, how to act according to their BP (Fig. 2) and the “monthly registration sheets” for a six month period in order to register their blood pressure twice a day, once in the morning and once in the evening (for the first seven days of each month) and to register contacts related to their BP (by phone, regular or urgent consultation at the office or hospital consultation) during that follow-up period.

#### Control group

Patients will be informed by their GP that they will continue their usual care regarding their BP and that they will be recalled to make an appointment with a member of the research team, who will provide them with information and basic recommendations for improvement of BP control and for completing the data corresponding to the baseline visit. Subsequently, members of the research team will deliver the “*Control group booklet”* containing the patient’s code, general information and basic recommendations for improving BP control, as well as the “monthly registration sheets” for a six month period in order to register contacts related to their BP (by phone, regular or urgent consultation at the general practice or hospital consultation) during the follow-up period.

Patients in the control and intervention groups will be informed that the research team will phone them four weeks after the baseline visit to clarify any doubts raised. If necessary, on-site visits will be arranged for further clarification.

**Fig. 3:**
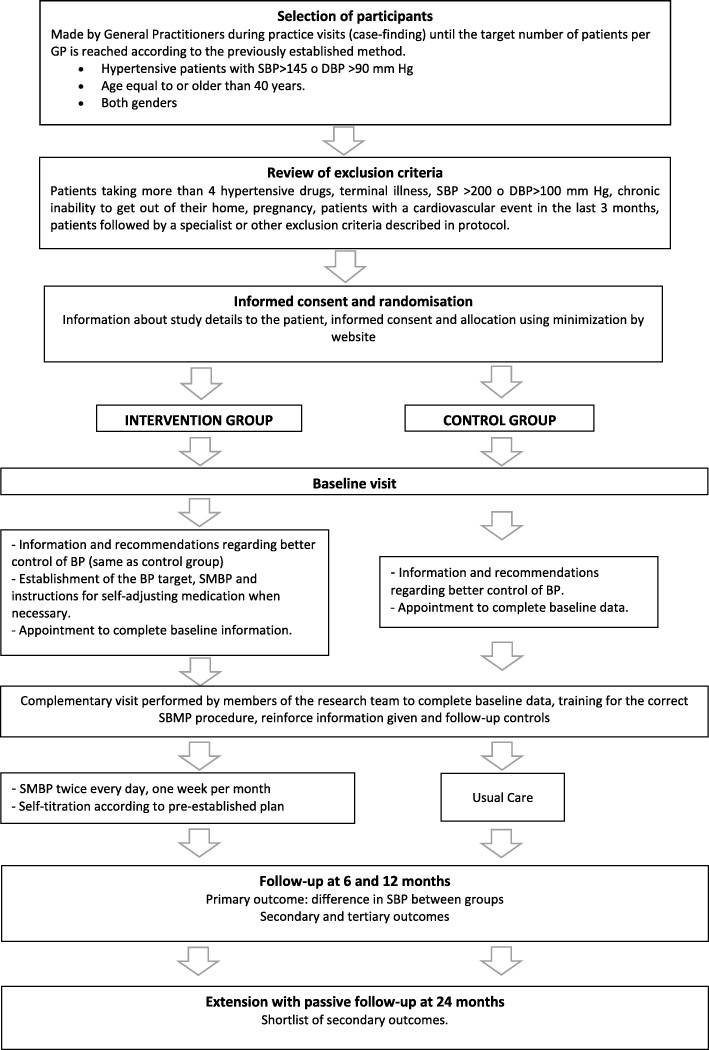
Participants’ flow through the study. *GP* General Practitioner, *SBP* Systolic Blood Pressure, *DBP* Diastolic Blood Pressure, *BP* Blood Pressure, *SMBP* Self-Management of Blood Pressure

Both groups will be contacted by phone at 3 months to clarify any doubts and at 6 months a follow-up visit will be established at the PCC, where the corresponding variables will be collected. The same will be done at 12 months. The follow-up variables will be collected up to a maximum of 6 weeks after the end of the follow-up period. An extension of the study will be performed with passive follow-up at 24 months, collecting a reduced set of outcome variables as secondary variables. Participants’ flow through the trial is outlined in Fig. 3.

Patients have the right to leave the study at any time. In addition, the researcher may discontinue a patient from the study if deemed necessary for any reason including: non-eligibility (retrospective if not detected at the time of inclusion, or prospective e.g. pregnancy during the follow up), an adverse event or disease progression involving incapacity to comply with trial procedures.

#### Sample size consideration

A sample size of 382 patients was estimated in order to have 90% power to detect a difference in SBP of 5 mmHg (SD 15 mmHg) between the intervention and the control group with a contrast of two-tailed hypotheses and an alpha error of 0.05. This figure represents a clinically relevant difference (which should represent a reduction of approximately 19% in strokes) and is in line with the results observed in previous trials in this field (TASMINH2 and TASMINH-SR) [15, 16]. These figures will be increased by 20% to compensate for possible drop-outs and follow-up loss, resulting in a total sample size of 458 participants.

### Intervention

#### Intervention group

##### Blood pressure self-monitoring

Patients will be trained to perform SMBP by the research team through a validated home blood pressure monitor (Omron model M3 HEM-7131-E). Patients will take their BP in the morning and in the afternoon, every day of the first week of each month. This will be done in the morning, before breakfast and before taking their medication (between 6 am. and 9 am.) and in the evening before dinner and before taking their medication (between 6 pm. and 9 pm.). These measurements will be recorded by the patients for the first seven days of each month on the “monthly registration sheets” located in the “*Intervention group booklet*”. If patients want to monitor their BP during the remaining weeks of the month, it is recommended that they just do so one day a week. Patients are instructed to act according to a table that contains easy-to-follow colour coded action steps. This guideline prompts the patient to contact the GP or visit the health center when BP values are very high or very low. Four or more above target readings in a month will require a change in medication (See Fig. 2).

##### Target blood pressure

Patients will be informed of their target BP, which will be established by their own GP and individualized for each patient based on the *Guidelines for the management of arterial hypertension of the European Society of Hypertension (ESH) and European Society of Cardiology (ESC)* [19]*.* Recommendations on target BP, according to cardiovascular risk and reflecting home as compared to office readings are shown in Table 1.

**Table 1:** Target blood pressure, according to cardiovascular risk conditions

Age	Clinical situation	TARGET BLOOD PRESSURE			
		SBP		DBP	
		SMBP	Office readings	SMBP	Office readings
< 80 years old	Without increased cardiovascular risk	≤135	≤140	< 85	< 90
	Diabetes	< 135	< 140	< 80	< 85
	Cerebrovascular disease (previous stroke or TIA)			< 85	< 90
	Coronary Heart Disease				
	Peripheral artery disease				
	Chronic kidney disease				
≥ 80 years old		< 145	< 150	< 85	< 90

*SBP* Systolic blood pressure, *DBP* Diastolic blood pressure, *SMBP* Self measured blood pressure

Modified from the *2013 ESH/ESC Guidelines for the management of arterial hypertension*. The European Society of Hypertension (ESH) and European Society of Cardiology (ESC)

##### Self-titration

In order to reach their target BP, each patient will be given a self-management plan to adjust medication if necessary depending on blood pressure measurements (See Fig. 2). The self-adjustment plan will consist of either an increase in the dose or additional medication. Therapeutic plan choice will be at the discretion of the GP, who will receive a copy of the Clinical Practice Guidelines of the European Society of Cardiology [19] to aid decision-making. If self-adjustment takes place, the participant will have an appointment with his/her GP within 3 weeks following self-adjustment, and a new tailored self-management plan will be provided.

#### Control group

Patients allocated to the control group will receive routine hypertension care with appointments and medication changes following the GP’s criteria in the context of routine clinical practice.

In both, the intervention and control group, all relevant concomitant care within usual clinical practice will be at the discretion of the GP.

### Outcomes

The primary outcome will be the change in mean SBP -mmHg- between baseline and 12 months.

Secondary outcomes will include:

1. Change in mean SBP at 6 and 24 months of follow-up.
2. Change in mean DBP at 6, 12, and 24 months of follow-up.
3. Percentage of patients with SBP < 140 mmHg and DBP < 90 mmHg at 6, 12 and 24 months of follow-up.
4. Quality of life (as measured by EuroQoL-5D) at 6, 12 and 24 months of follow-up.
5. Adherence measured by proportion of days covered (PDC) at 6 and 12 months of follow-up.
6. Persistence, defined as period of continuous use of the corresponding drug from the beginning of the follow-up until its discontinuation at 6 and 12 months of follow-up.
7. Therapeutic inertia (TI), defined as the number of patients whose pharmacological treatment had not been modified, divided by the number of patients not reaching the target values (SBP and/or DBP measurements taken at 6 and 12 months of follow-up), ​​according to the recommendations of the European Society of Hypertension and European Society of Cardiology [19].

Other outcome measures:

1. Changes in lifestyle (smoking, exercise, body weight) at 6, 12, and 24 months compared to these characteristics at baseline.
2. Clinical events: We will assess if any of the following adverse events are present during the follow-up: angina, myocardial infarction, stroke, hypotensive crisis and death.
3. Use of health services for hypertension at 6, 12 and 24 months.
4. Incremental cost per quality-adjusted life year gained in the intervention group compared to the control group.
5. Views and experiences of patients and health professionals on the self-management (SMBP plus self-titration) of hypertension.

### Data collection

Data will be collected at the different participant study sites. Details on type of data and timing of collection are shown in Fig. 1.   

Data entry, coding, security, and storage, including any related processes to promote data quality (eg, double data entry, etc) and other aspects related to data management such as data monitoring of the ADAMPA study, will be performed by the SCReN platform (for more information on the Screen platform and its role regarding the ADAMPA study, see: https://www.scren.es/, and https://www.scren.es/proyectos.php).

### Statistical analysis

Analysis will be on an intention-to-treat basis for complete cases. We will use mixed models (general linear modeling –GLM) to compare SBP at 12 months between the intervention and control groups. This analysis will be presented in both crude and adjusted forms for the different covariates of interest (baseline BP, gender, GP/PCC-random effect, diabetes, etc.). A sensitivity analysis will be performed to examine the potential effect of missing data, which will include substitution by multiple imputation, replacement of data lost by the most recent data or by the mean of the series. Additionally, analyses of the main outcome measure by subgroups of age, gender, comorbidity, level of chronicity, better control at baseline, etc. will be performed.

Differences in secondary outcome measures (DBP, percentage of patients controlled, PDC, persistence and TI) will be analyzed using methods similar to those used for analysis of the main outcome measure.

### Economic analysis

The economic analysis will include a cost-consequence analysis, estimating both the costs (hospitalizations, outpatient visits, emergency visits and antihypertensive drugs) and the potential benefits (e.g. reduced incidence of stroke, myocardial infarction, etc.) in natural units. In addition, we will collect information on Health-Related Quality of Life (HRQOL) through the EQ-5d questionnaire, which will allow us to obtain utilities and therefore perform a cost-utility analysis with the estimated benefits in terms of Quality-Adjusted Life-Years (QALY).

A modeling will be performed to obtain longer-term predictions of the results observed in the trial. The results on which this modeling will be based will be survival, quality of life and costs associated with clinical events. A sensitivity analysis (deterministic and probabilistic) will be performed to analyze the robustness of the results. Key parameters will be modified to determine their impact on results. All analyses will be performed using STATA version 14.

### Quality sub-study

Qualitative research techniques will seek to provide an in-depth understanding of the positive elements and areas of improvement related to self-titration and self-monitoring intervention. To this end, two meetings will be held, one with professionals (GPs and nurses) and one with patients, using the Nominal Group Technique (NGT). The NGT is a working methodology that establishes a framework for highly structured interaction that enables participation and equal consideration of the contributions of all members of the working group, and allows the identification of priorities, consensus and disagreement, solution generation and decision-making in an agile and objective manner [25].

## Discussion

The ADAMPA trial is a clinical research project that aims to improve the control of BP through training the patient for self-management of their hypertension. Hypertension is a risk factor of high prevalence that, even today, presents an unacceptable percentage of uncontrolled patients, according to the recommendations of the guidelines of clinical practice for BP control.

If the data from this trial show positive results, the study may contribute to a change of strategy in the treatment of hypertension, focusing on the patient as the main actor to achieve these objectives. Furthermore, this approach might contribute to the financial sustainability of the National Health Service.

## Abbreviations

BP: Blood pressure
DBP: Diastolic blood pressure
GP: General practitioner
mm Hg: Millimeter of mercury
NGT: Nominal Group Technique
PC: Primary care
PCC: Primary care centers
PDC: Proportion of days covered
SBP: Systolic blood pressure
SD: Standard deviation
SMBP: Self-measured blood pressure monitoring at home
TI: Therapeutic inertia
